# The purinergic receptor P2X7 as a modulator of viral vector-mediated antigen cross-presentation

**DOI:** 10.3389/fimmu.2024.1360140

**Published:** 2024-04-22

**Authors:** Ylenia Longo, Sara Moreno Mascaraque, Giuseppe Andreacchio, Julia Werner, Ichiro Katahira, Elena De Marchi, Anna Pegoraro, Robert Jan Lebbink, Karl Köhrer, Patrick Petzsch, Ronny Tao, Francesco Di Virgilio, Elena Adinolfi, Ingo Drexler

**Affiliations:** ^1^ Institute of Virology, Universitätsklinikum Düsseldorf, Düsselorf, Germany; ^2^ Institute of Molecular Medicine II, Universitätsklinikum Düsseldorf, Düsseldorf, Germany; ^3^ Department of Medical Sciences, University of Ferrara, Ferrara, Italy; ^4^ Institute of Infection Immunity, University of Utrecht, Utrecht, Netherlands; ^5^ Biological and Medical Research Center (BMFZ), Medical Faculty, Heinrich-Heine-University, Düsseldorf, Germany

**Keywords:** Modified Vaccinia Virus Ankara, cross-presentation, P2RX7, extracellular vesicles, cytokines

## Abstract

**Introduction:**

Modified Vaccinia Virus Ankara (MVA) is a safe vaccine vector inducing long- lasting and potent immune responses. MVA-mediated CD8^+^T cell responses are optimally induced, if both, direct- and cross-presentation of viral or recombinant antigens by dendritic cells are contributing.

**Methods:**

To improve the adaptive immune responses, we investigated the role of the purinergic receptor P2X7 (P2RX7) in MVA-infected feeder cells as a modulator of cross-presentation by non-infected dendritic cells. The infected feeder cells serve as source of antigen and provide signals that help to attract dendritic cells for antigen take up and to license these cells for cross-presentation.

**Results:**

We demonstrate that presence of an active P2RX7 in major histocompatibility complex (MHC) class I (MHCI) mismatched feeder cells significantly enhanced MVA-mediated antigen cross-presentation. This was partly regulated by P2RX7-specific processes, such as the increased availability of extracellular particles as well as the altered cellular energy metabolism by mitochondria in the feeder cells. Furthermore, functional P2RX7 in feeder cells resulted in a delayed but also prolonged antigen expression after infection.

**Discussion:**

We conclude that a combination of the above mentioned P2RX7-depending processes leads to significantly increased T cell activation via cross- presentation of MVA-derived antigens. To this day, P2RX7 has been mostly investigated in regards to neuroinflammatory diseases and cancer progression. However, we report for the first time the crucial role of P2RX7 for antigen- specific T cell immunity in a viral infection model.

## Introduction

The P2X7 receptor (P2RX7) belongs to the ionotropic purinergic P2X subfamily and is mostly expressed in immune, endothelial and epithelial cells (1, 2). High concentrations of adenosine triphosphate (ATP) are known to activate the ion channel as a danger-associated molecular pattern (DAMP), leading to the intracellular increase of Na^+^ and Ca^2+^ and the efflux of K^+^. P2RX7 has been shown to be crucial for the regulation of various signaling pathways, such as the inflammasome pathway or those that lead to the release of cytokines, cell death or mitochondrial activation (3–6). Next to its contribution to various pathological diseases, P2RX7 activation is also associated with the release of extracellular vesicles (EVs) or particles (EPs) (7–9). They contain various immunostimulatory molecules known to be pivotal for the activation of antigen presentation processes (10, 11). Recently, functional P2RX7 expression has been linked to increased viral loads of human herpes virus 6A (12). Furthermore, data from our lab (13, 14) indicate increased expression of the P2X7 receptor during infection with Modified Vaccinia Virus Ankara (MVA).

MVA is a highly attenuated double-stranded DNA virus belonging to the family of the *Poxviridiae* and the genus *Orthopoxvirus* (15–17). For the generation of MVA, the parental strain Chorioallantois Vaccinia Virus (CVA) was passaged over 570 times in chicken embryo fibroblasts, leading to six large deletions in the MVA genome and the inability to replicate in most mammalian cells (17–20). Since MVA fails to generate infectious particles in humans, it has been developed as a suitable vector for vaccine design (21–23). It is able to express a large amount of recombinant DNA, and it induces strong humoral and cellular immune responses upon vaccination (22, 24).

Interestingly, robust and long-lived cytotoxic T cell (CTL) immunity is dependent on cross-presentation during MVA infection (25, 26). Upon infection with MVA, cells undergo apoptosis, containing and releasing antigens to be phagocytosed by professional antigen-presenting cells (APCs) (27, 28). Upon internalization of the antigen, two distinct pathways can lead to the loading of MHC class I molecules (MHCI): the vacuolar and the cytosolic antigen-processing pathway (29). A peptide-MHCI-complex can either be generated by TAP interacting with internalized phagosomes containing the peptide to be processed or by processing already internalized peptides via the endoplasmic reticulum (30). In the vacuolar pathway, the processing and loading, both will occur in the vacuoles themselves (31). The preformed MHCI-peptide complex is then released and exported to the cell surface where CTL can be activated and release inflammatory cytokines, such as IFNү and TNFα (32). Since cross-presentation is essential for optimal CD8^+^ T cell priming for various pathogens as well as vector delivery systems, its molecular regulation has encouraged intense investigations. More evidence suggests that the stimulus for successful cross-presentation does not originate from the non-infected antigen-presenting cell, but rather from the bystanding initially infected cell, which we term feeder cell. We have recently shown that STING in feeder cells is involved in regulating CD8^+^ T cell responses via type I interferon production acting on the cross-presenting APC (33).

In this study, we aim to analyze the role of other innate triggers in feeder cells for MVA-induced antigen cross-presentation. The innate immune system serves as the first line of defense once a pathogen is encountered and P2RX7 as a member of the innate system has been shown to be potently activated by extracellular ATP, which is released by different stimuli. ATP is essential during vaccinia virus infection and therefore for the regulation of immune responses (34, 35). P2RX7 has been described to be involved in antigen presentation (36). It alters the secretome in cells bearing the active P2RX7, such as the production of extracellular vesicles that might contain antigens or the production and release of varying inflammatory cytokines and chemokines (10, 37, 38). Additionally, P2RX7 activity has been associated with the expression of *Nfatc1*, belonging to the group of primary response genes modulated by immune signals (39–41). Therefore, we investigated the involvement of the P2X7 receptor, as a member of the innate immune system, in infected feeder cells during cross-presentation of MVA-derived antigens.

The regulation of cross-presentation has been intensively studied for years, however, detailed knowledge about the molecular mechanisms that underlie the relevant pathways as well as about the innate triggers to initiate the process in the cross-presenting APC is lacking. In the present study, we aimed to investigate the potential role of P2RX7 as an innate stimulus in infected feeder cells for the initiation and modulation of cross-presentation in the non-infected bystander APC. We show that the ATP-sensitive P2RX7 from the BALB/c strain in feeder cells is essential for enhancing CD8^+^ T cell responses via cross-presentation *in vitro*. We show that various P2RX7-dependent pathways that we analyzed and which are crucial for the initiation of immune responses, such as the release of inflammatory cytokines, mRNA and the presence of apoptotic stimuli, were modulated in the presence of functional P2RX7 in infected feeder cells. Our findings suggest that the improved cross-presentation capacity of antigen-presenting cells co-cultured with infected feeder cells bearing active P2RX7 might be due to the activation of several pathways in feeder cells that may act together to orchestrate the immune response. To our knowledge, this is the first report instigating the function of the P2RX7 for regulation of MVA-mediated T cell immunity.

## Results

### The plasma membrane P2X7 receptor is not functional in Cloudman (CM) cells and reconstitution with active P2RX7 from BALB/c mice enhances the release of extracellular particles after MVA infection

**Figure 1:**
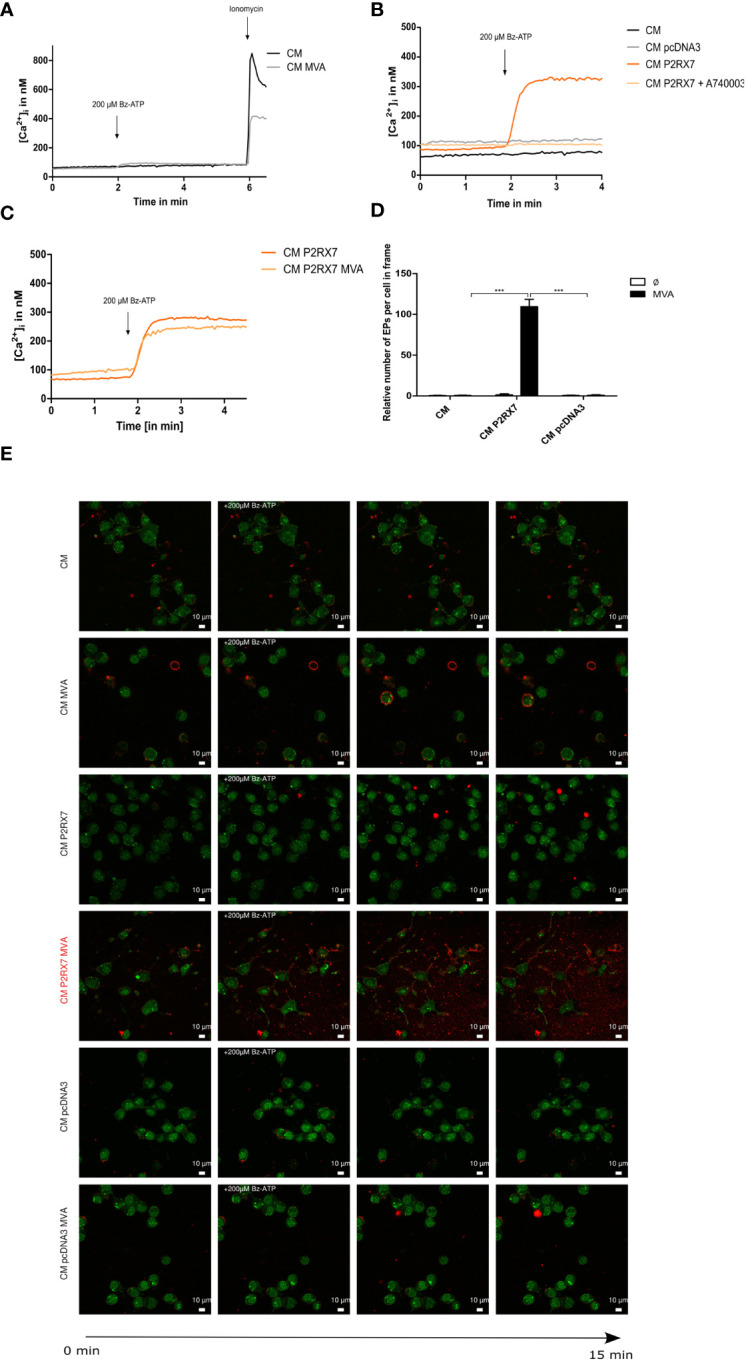
The P2X7 receptor is inactive in Cloudman (CM) cells (DBA background) and transfection with functional P2RX7 from BALB/c mice restores P2RX7-specific functions. **(A)** Fluorometric analysis of intracellular Ca^2+^ (iCa^2+^) influx as a known marker of P2RX7 activity to demonstrate the function of the plasma membrane-located P2RX7 in CM cells infected with MVA or mock. Activity of P2RX7 in CM wildtype (WT) cells infected either with MVA-PK1l-Ova (20hpi/MOI1) expressing ovalbumin under the control of the vaccinia virus early promoter PK1L or mock-infected was investigated by measurement of intracellular Ca^2+^ concentrations of FURA-2-AM loaded cells upon stimulus with 200µM Bz-ATP. **(B)** CM cells were transfected with empty vector (CM pcDNA3) or P2RX7 containing plasmid DNA. Activity was assessed at least one week post transfection by fluorometric assay. CM P2RX7 cells were additionally pre-treated for 5min with 20µM A740003 (CM P2RX7 A740003), a P2RX7 specific inhibitor. **(C)** Intracellular calcium concentrations were further tested in CM P2RX7-transfected cells 20h post-MVA infection (MOI1) (CM P2RX7 MVA) or mock-infected cells (CM P2RX7). **(D)** Quantification of extracellular particles per total number of cells was started after addition of 200µM Bz-ATP for a time frame of approximately 30sec (three subsequent frames) from **(E)** MVA- (MOI1) or mock-infected CM WT or transfected cells (CM P2RX7 or CM pcDAN3) cells. Cells were stained with quinacrine nucleic acid stain and PKH26 membrane stain and then stimulated with 200µM Bz-ATP to visualize the release of particles using confocal image analysis. Data shown, represent one from at least n=3 **(A–C)** or n=2 independent experiments **(D, E)**. Statistical significance (P) ***P ≤ 0.001.

In line with the literature (42) and our sequencing data (**Supplementary Figure 1A**), the fluorometric analysis of intracellular Ca^2+^ influx failed to demonstrate activation of the plasma membrane-located P2RX7 in Cloudman (CM) mock-or MVA-PK1L-Ova infected cells upon Bz-ATP specific stimulus (**Figure 1A**). Although recent New Generation Sequencing analysis (14) has shown that the expression of the P2X7 receptor was upregulated after MVA infection, qRT-PCR analysis of infected CM cells failed to demonstrate an upregulation of expression but indicated a stable constitutive expression after infection (**Supplementary Figure 1B**). For the matter of simplification, we distinguish between active and inactive P2RX7, referring to the BALB/c P2RX7 or DBA/C57BL/6 P2RX7 (less sensitive to ATP stimuli (42)), respectively. To exclude that the lack of intracellular Ca^2+^ increase was due to faulty loading of the fluorescent indicator FURA-2-AM, we additionally stimulated the cells with ionomycin, which is a receptor-independent trigger for a maximal increase of intracellular Ca^2+^. Even higher Bz-ATP stimuli could not increase intracellular Ca^2+^ concentrations (**Supplementary Figure 1C**). Interestingly, when stimulating the CM cells with ATP, known to activate additional purinergic receptors besides P2RX7, we observed a gain in intracellular Ca^2+^ amounts, suggesting the activity of other receptors of this or the P2Y receptor family (43) (**Supplementary Figure 1D**). Next, we were interested in studying the role of active P2RX7 and transfected our CM cells with the fully functional P2RX7 expressed by BALB/c mice (42). We were able to demonstrate its activity after transfection, by an increase in the concentration of intracellular Ca^2+^ upon Bz-ATP stimulus (**Figure 1B**). This response was reversed when the reconstituted cells were treated with the P2RX7-specific competitive inhibitor A740003 at 20µM. Transfection with the empty vector control, similar to CM WT cells, did not alter the amount of intracellular Ca^2+^ upon Bz-ATP treatment. Toxicity of A740003 was excluded (**Supplementary Figure 1E**). Furthermore, the receptor activity represented by an increased Ca^2+^ influx in the P2RX7-transfected cells appeared to be significantly higher at earlier time points during MVA infection after 4 h.p.i. (**Supplementary Figure 1F**), suggesting that the infection itself modulates the activity of the receptor. However, calcium levels did not change after 20h MVA infection when compared to uninfected cells indicating that the receptor activity is only transiently increased after infection (**Figure 1C**). Interestingly, we did not observe P2RX7-specific pore function in P2RX7 transfected cells (**Supplementary Figure 1G**) (44). The release of extracellular particles, which is reported to be partly P2RX7 dependent (7), was enhanced when P2RX7 transfected cells were infected with MVA, supporting the regulation of P2RX7-specific functions during MVA infection (**Figures 1D, E**). Overall, we confirmed the reconstitution and functionality of P2RX7 in CM cells after transfection.

### Active P2RX7 in feeder cells promotes MVA antigen cross-presentation

**Figure 2:**
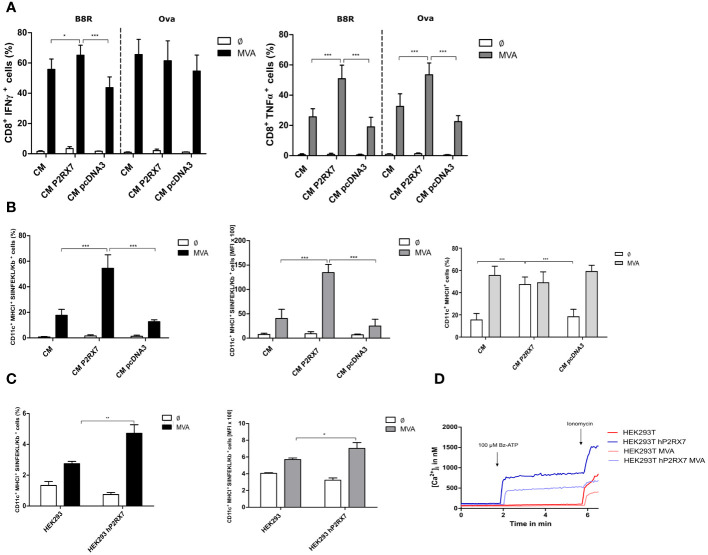
Antigen cross-presentation is enhanced in the presence of active P2RX7 in CM feeder cells. GM-CSF bone marrow derived-dendritic cells (BMDCs) were co-cultured with CM WT (CM), control plasmid (CM pcDNA3) P2RX7 transfected (CM P2RX7) feeder cells infected with MVA-PK1L-Ova expressing ovalbumin under control of the viral early promoter PK1L (MVA) at MOI1 or mock-infected (Ø). At 20hpi, B8R- or Ova-specific CD8^+^ T cells were added to the co-culture for 4h. Presence of intracellular activation markers was determined by flow cytometric analysis (FACS) of **(A)** IFNү (left) or TNFα (right) production. **(B)** Frequency (left) and mean fluorescent intensity (MFI) (middle) of SIINFEKL/H2-K^b^ surface expression (FACS). MHCII (%) expression in BMDCs (right). **(C)** SIINFEKL/Kb expression was also assessed in BMDC that had been co-cultured with HEK293 cells (WT or hP2RX7 transfected) infected with either mock (Ø) or MVA-PK1L-Ova (MVA) at MOI1 for 20h. **(D)** Fluorometric analysis of intracellular calcium concentrations in HEK293 WT (HEK293) and hP2RX7 transfected (HEK293 hP2RX7) cells with or without MVA-PK1L-OVA (MOI1, 20h) infection and 200µM Bz-ATP stimulus. Data are pooled from at least n=3 independent experiments (n=3-5) and shown as means ± SD. P values indicate statistical significance (P) with *P ≤ 0.05 **P ≤ 0.01; ***P ≤ 0.001.

Recent studies have shown that innate triggers derived from infected feeder cells are relevant for the activation of T cells by antigen-presenting cells (33). We were interested in investigating whether the presence of a functional P2X7 receptor in feeder cells may have an impact on the antigen uptake and presentation capacity of bone marrow-derived dendritic cells (BMDCs) for activation of CD8^+^ T cells. We demonstrate that using MVA-OVA-infected feeder cells bearing the active P2X7 receptor led to a significantly higher CD8^+^ T cell activation as determined by IFNү production in B8R- specific T cells or by TNFα production in either B8R- or OVA-specific T cell lines when co-cultured with uninfected dendritic cells as cross-presenting APC (**Figure 2A**). These data are in line with the P2RX7-dependent release of these cytokines in mice (45). The frequency of cross-presenting BMDCs with SIINFEKL/H2-K^b^ complexes on the cell surface, as well as the amount of these peptide/MHCI complexes per cell, was significantly increased (**Figure 2B** left, middle). Interestingly, the presence of the active P2RX7 in CM cells led to the increase of MHCII surface expression in co-cultured BMDCs (**Figure 2B** right). The expression of other maturation markers, such as CD40 or CD86 on co-cultured BMDCs was not affected (data not shown). Furthermore, pre-treatment of CM P2RX7 cells with A740003 before co-culture with BMDCs led to a significantly reduced CD8^+^ T cell activation (**Supplementary Figure 2A**) and SIINFEKL/H2-K^b^ expression (**Supplementary Figure 2B**) which was comparable to CM WT or CM pcDNA3 cells. To corroborate the specific function of P2RX7, we used HEK293 as feeder cells expressing active human P2RX7 (**Figure 2D**). Indeed, the co-incubation of BMDCs with MVA-infected HEK293 hP2RX7 feeder cells resulted in increased SIINFEKL/H2-K^b^ expression (**Figure 2C**). In sum, we demonstrated that the presence of functional P2RX7 in feeder cells aids in improving antigen cross-presentation upon MVA infection.

### Functional P2RX7 does not alter antigen availability or replication capacity of MVA but impacts the gene expression of viral antigens

**Figure 3:**
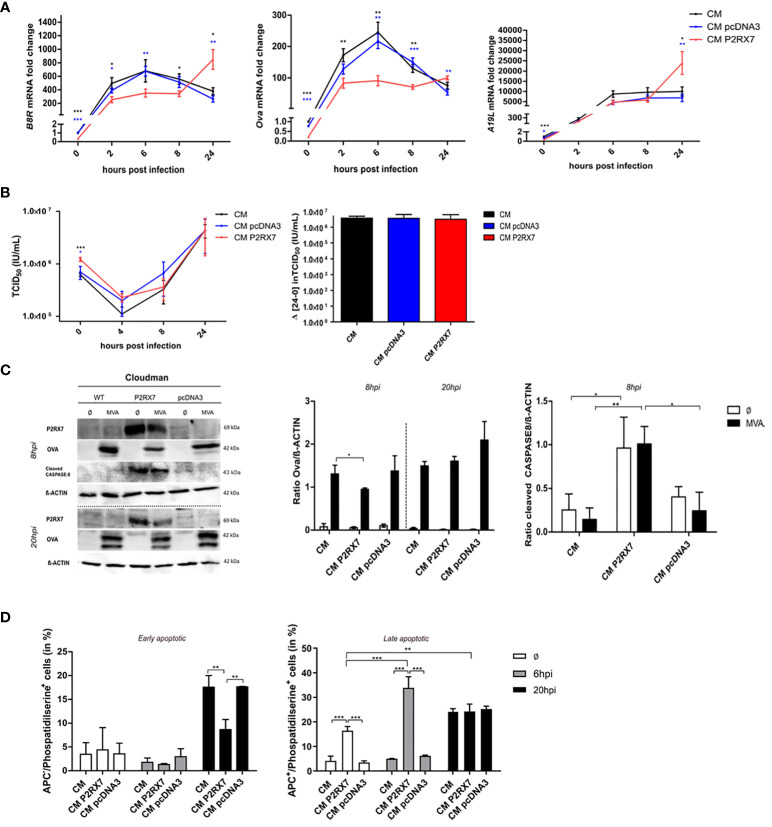
Expression of active P2RX7 in feeder cells alters several signaling pathways that may be correlated with enhanced antigen availability and cross-presentation by BMDCs. **(A)** CM WT (CM) or pcDNA3 or P2RX7 transfected cells (CM pcDNA3 or CM P2RX7) were infected with MVA-PK1L-Ova at MOI1 for 0h to 24h to assess the expression of viral antigens B8R and A19L or recombinant antigen Ova, respectively. mRNA fold change is shown as expression of the respective gene at each time point compared to the 0h time point of CM WT cells. **(B)** Viral growth kinetics of MVA-p7.5-GFP expressing green fluorescent protein (GFP) under control of the viral early/late promoter p7.5. (Left) Wildtype or transfected CM cells were infected for 0h to 24hpi and viral titers were determined by back titration on DF1 cells at the indicated hpi as tissue culture infectious dose (TCID_50_). (Right) Final viral loads after 24hpi were measured by subtracting viral output at 24hpi with viral input at 0hpi. **(C)** Western Blot analysis of WT or transfected CM cells upon MVA-PK1L-Ova infection at 8hpi or 20hpi (left). Quantification of OVA (middle) or cleaved CASPASE-8 (right) protein amounts in cellular extracts. **(D)** Phosphatidylserine residues on the cell surface of mock- (Ø) or MVA-infected (MOI1) WT or transfected CM cells at either 6hpi or 20hpi. Quantification of either APC-negative (APC-) (live/dye non-permissive) (left) or APC-positive (APC+) (dead/dye permissive) (right) within phosphatidylserine positive cells, depicting either early or late apoptotic cells, respectively. Experiments are shown as means with SEM **(A)** or SD of n=3 independent biological replicates with statistical significance (P) *P ≤ 0.05; **P ≤ 0.01; ***P ≤ 0.001.

We first hypothesized that the increased SIINFEKL/H2-K^b^ surface expression on antigen-presenting cells and the improved CD8^+^ T cell activation that we found when BMDCs were co-cultured with P2RX7 feeder cells, might be due to an increased amount of viral antigens. We analyzed the expression of early antigens, such as *B8R*, a native MVA antigen, or *Ova*, expressed under the control of the early MVA promoter PK1L, or the late viral antigen *A19L* in CM P2RX7 or CM pcDNA3 transfected cells and compared it to CM WT cells. Expression of these antigens was initially lower in CM P2RX7 cells compared to CM WT or the empty vector control CM pcDNA3. However, at 24hpi *B8R*, *Ova* and *A19L* mRNA fold change in CM P2RX7 cells was significantly higher when compared to CM WT or CM pcDNA3 cells (**Figure 3A**). We concluded that the expression kinetics of viral genes in CM P2RX7 was delayed, although mRNA expression at later time points in these cells was significantly higher. This was further confirmed when analyzing the replication capacity of MVA in the different CM feeder cell lines. We also observed a higher residual viral titer at 0hpi in CM P2RX7 (**Figure 3B**), suggesting a possible role of the P2X7 receptor for viral entry, as previously stated (12). Since MVA has lost its ability to replicate in most mammalian cells (19), we wanted to exclude the possibility that the presence of this receptor was affecting viral replication behavior and, as a consequence, promote antigen cross-presentation. However, at 24hpi viral particle amounts in CM P2RX7 cells were comparable to CM WT or CM pcDNA3 cells (**Figure 3B** right). Similarly, the synthesis of OVA protein in CM P2RX7 cells was initially reduced at 8hpi but comparable at 20hpi (**Figure 3C**), indicating that expression of viral genes and synthesis of corresponding proteins might be delayed in CM P2RX7 transfected cells.

The above data exclude that altered protein amounts in feeder cells as the source of antigenic uptake by antigen-presenting cells contributed to the improved antigen presentation by BMDCs co-cultured with infected CM P2RX7 cells. It has been postulated that the antigen has to be released but needs to be still cell-associated for the antigen-presenting cells to phagocytose and process it for cross-presentation to CD8^+^ T cells (46–48). We confirmed the activation of the extrinsic apoptosis pathway by cleavage of Caspase 8 (**Figure 3C**). The exposure of phosphatidylserine residues on the cell surface is important for the activation of phagocytosis by APCs and has been linked to P2RX7 activation (49–51). We demonstrate that the presence of the functional P2X7 receptor led to the increased surface expression of phosphatidylserine residues in cells having a permeable cell membrane (**Figure 3D** right) when mock-infected or infected with MVA for 6h. In contrast, MVA-infected CM P2RX7 with intact cell membrane, hence alive cells, displayed reduced phosphatidylserine levels as compared to CM WT or CM pcDNA3 cells after 20h MVA infection. Overall, we speculate that expression of the functional P2X7 receptor in CM feeder cells does not modulate the antigen-presentation capacity by APCs by increasing the total amount of antigenic protein in feeder cells, but rather allows for increased viral mRNA levels in infected cells at later time points as well as by altering apoptotic pathways. This depicts the importance of further analyses on the RNA level and the possible altered localization of P2RX7 affecting other cellular pathways.

### Extracellular particles as well as supernatant released from feeder cells with active P2RX7 promote MVA antigen cross-presentation

**Figure 4:**
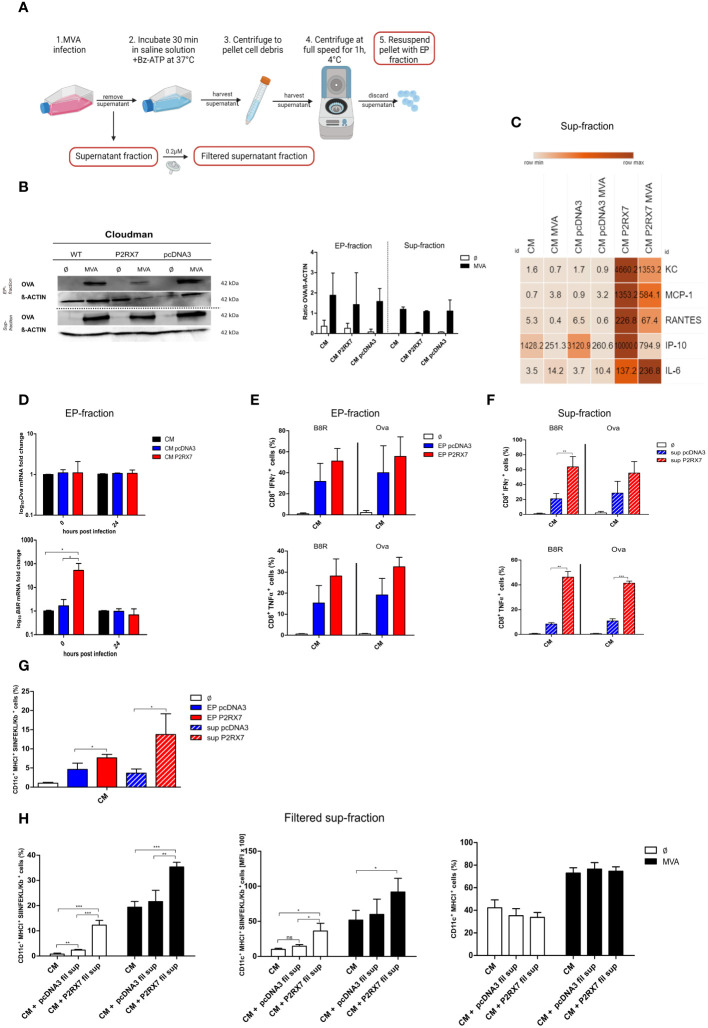
Subcellular fractions of P2RX7 transfected CM feeder cells enhance MVA-mediated antigen cross-presentation upon co-cultivation with cross-presenting BMDCs. **(A)** Experimental setup to extract extracellular particle (EP) fraction and supernatant fraction (sup) from infected cells. Figure created in Biorender with permission. **(B)** Both fractions were analyzed for OVA protein content at 20 hours post MVA-PK1L-Ova infection (MVA) at MOI1.5 (EP) or MOI1 (sup). **(C)** Expression and release of inflammatory chemokines was assessed in the supernatant fraction of CM, CM pcDNA3 or CM P2RX7 cells that were either mock- or MVA-infected (MOI1 for 20h) by Legendplex assay. **(D)** mRNA expression. Quantification of Ova (upper) or B8R mRNA (lower) at 0hpi and 20hpi in EP-fractions of MVA-PK1L-OVA infected cells (MOI1.5). **(E, F)** EP- or sup-fractions from MVA-PK1L-Ova infected CM pcDNA3 or CM P2RX7 cells that were added to the co-culture of uninfected CM WT feeder cells with uninfected BMDCs which were then co-cultured with B8R- or Ova-specific CD8^+^ T cells for 4h to assess IFNү or TNFα production upon antigen-specific activation. **(G)** SIINFEKL/H2-K^b^ surface expression of BMDCs was determined after adding either the EP- or the sup-fraction from control (pcDNA3) or P2RX7 transfected (P2RX7) cells to uninfected BMDCs co-cultured with uninfected CM WT (CM) cells. **(H)** SIINFEKL/H2-K^b^ surface expression of BMDCs by frequency (left) or mean fluorescence intensity (middle) after the addition of filtered supernatants from MVA-infected CM pcDNA3 or CM P2RX7 cells to either mock-infected (Ø) or MVA-PK1L-OVA (MVA) at MOI1 infected CM cells. (Right) MHCI expression (total H2-K^b^) of BMDCs was assessed as a control. Plots show the mean of n=3 independent experiments with SD and statistical significance (P) *P ≤ 0.05; **P ≤ 0.01; ***P ≤ 0.001. ns, not significant.

Extracellular particles are known to contain crucial regulatory molecules for cell-to-cell communication (11). Similarly, supernatant from P2RX7 transfected cells differs from control cells (37). Here we hypothesized that both the extracellular particle fraction (EP-fraction), as well as the supernatant fraction (sup-fraction) from CM P2RX7 cells, are responsible for the enhanced antigen cross-presentation we observed. We first isolated the EP-fraction and the supernatant fraction as depicted in **Figure 4A** and determined both, mRNA and protein content. OVA protein amounts were comparable in wildtype and transfected CM cells after overnight infection in both, the EP- and the sup-fraction (**Figure 4B**). Interestingly, mRNA levels of the MVA early antigen *B8R*, but not *Ova* or *A19L* expression, were significantly higher in the EP-fraction at 0 hours post-infection leading to the hypothesis that the lower mRNA expression we observed in the cell extracts (**Figure 3A**) might be due to the release of RNA in extracellular particles (**Figure 4D**). This finding highlights the importance of focusing on the role of RNA in the regulation of MVA antigen presentation. We also showed that CM P2RX7 transfected cells allowed the secretion of significantly higher amounts of inflammatory cytokines pre- as well as post-infection (**Figure 4C**), demonstrating that the presence of functional P2RX7 modulates other signaling pathways as well. A detailed overview of the secreted cytokines can be seen in **Supplementary Figure 3A**. To further investigate the function of these culture sub-fractions from P2RX7 transfected cells, we delivered EP- and sup-fractions to the co-culture of uninfected CM cells and BMDCs to monitor subsequent CD8^+^ T cell activation and expression of SIINFEKL/H2-K^b^ complexes on dendritic cells. The EP-fraction from CM P2RX7 was not able to induce stronger CD8^+^ T cell IFNү or TNFα production as compared to the EP-fraction derived from pcDNA3 transfected CM cells. However, the sup-fraction led to increased IFNү and TNFα production when added to the co-culture of CM and BMDCs (**Figure 4F**). Interestingly, both fractions from CM P2RX7 cells were able to induce a significantly higher SIINFEKL/H2-K^b^ expression as compared to the empty vector control-derived fractions (**Figure 4G**). To establish whether these fractions could initiate similar CD8^+^ T cell activation as the infection of CM P2RX7 cells, these were added to MVA-infected CM WT cells (**Supplementary Figures 3B–E**). Even though both fractions seem to contribute to the increased expression of SIINFEKL/H2-K^b^ on BMDCs, the addition of EPs or supernatants from infected P2RX7 CM cells could not further increase CD8^+^ T cell activation or SIINFEKL/H2-K^b^ expression on BMDCs significantly. In addition, we filtered supernatants from infected feeder cells to remove any cell components larger than 0.2µM, such as apoptotic bodies (but leaving exosomes and microvesicles in the fraction) and added this filtered supernatant (fil sup) to either uninfected CM or MVA-infected cells (**Figure 4H** left, middle). We found a significant contribution of the filtered supernatant from CM P2RX7 cells for the activation of dendritic cells and subsequent presentation of the SIINFEKL peptide on its MHCI. The total amount of cross-presenting BMDCs (frequency) with SIINFEKL/H2-Kb complexes at the cell surface as well as the amount of SIINFEKL/H2-Kb complexes per BMDC (MFI) was significantly increased (**Figure 4H** left, middle). Notably, MHC I expression (total H2-K^b^) of the antigen-presenting cells was not altered upon the addition of this filtered supernatant (**Figure 4H** right). To sum this up, we demonstrate that the cellular fraction of P2RX7 transfected cells as well as other subcellular fractions are critically involved in enhancing antigen cross-presentation.

### The presence of active P2RX7 modulates mitochondrial function

**Figure 5:**
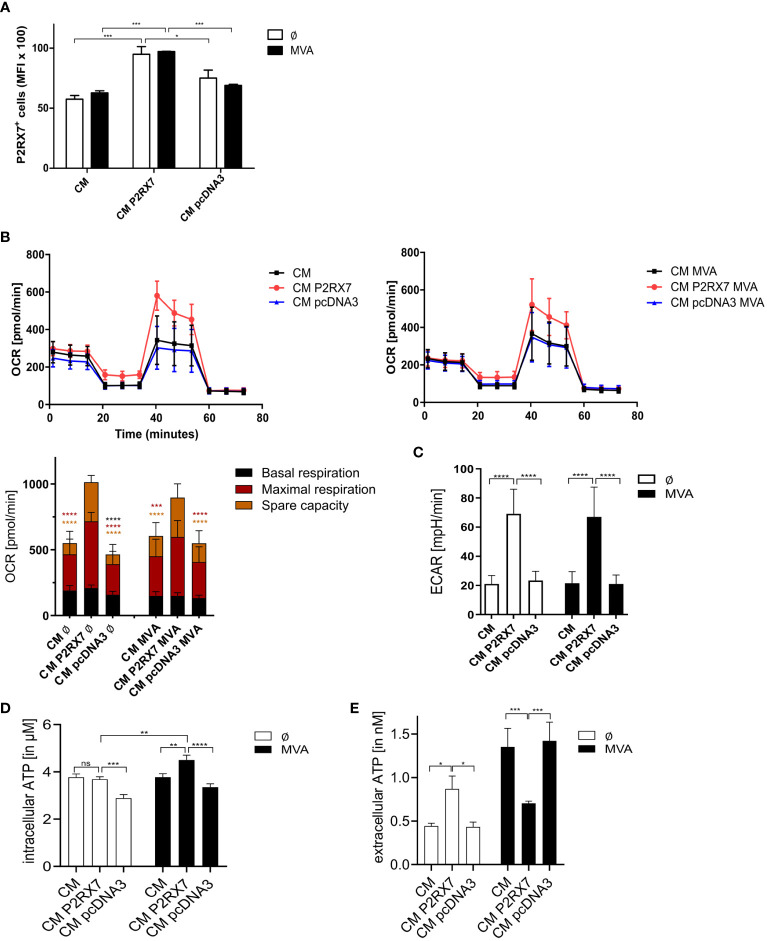
Presence of functional P2RX7 affects mitochondrial functions. **(A)** Intracellular expression of P2RX7. Mean fluorescent intensities (MFI) in mock- (Ø) or MVA-infected (MOI1, 20hpi) (MVA) CM WT (CM), empty vector control (CM pcDNA3) or P2RX7 (CM P2RX7) transfected cells. **(B)** Mitochondrial activity was assessed by measurement of the oxygen consumption rate (OCR) after treatment of CM WT, CM pcDNA3 or CM P2RX7 transfected cells with modulators of the electron transport chain such as Oligomycin, FCCP, and Rotenone/Antimycin A) (upper graphs) with (right) or without MVA infection (left) according to the Mitostress test kit (Seahorse). (lower graph) Mock-infected or MVA-infected (MOI5 for 6h) CM WT, CM pcDNA3, or CM P2RX7 cells were used to determine basal and maximal respiration as well as spare capacity upon addition of above mentioned modulators. **(C)** Comparable conditions were used to assess the extracellular acidification rate (ECAR) as an indicator for glycolysis processes in the indicated mock or MVA-infected (MOI5 for 6h) cells. **(D)** Quantification of intracellular or **(E)** extracellular ATP in CM WT, CM pcDNA3, or CM P2RX7 transfected cells either mock or MVA-infected (MOI5 for 6h). Data shown are of at least n=3 independent experiments, depicted as means with SD or SEM **(D, E)** and statistical significance (P) *P ≤ 0.05; **P ≤ 0.01; ***P ≤ 0.001; ****P ≤ 0.0001. ns, not significant.

As a plasma membrane receptor, P2RX7 is associated with the activation of the canonical inflammasome pathway (52). Recent studies, however, show that P2RX7 can also localize to and function intracellularly on mitochondrial structures (53). We confirmed the presence of intracellular P2X7 receptors in our feeder cells. These were significantly increased in P2RX7 transfected CM cells (**Figure 5A**). Mitochondria and energy metabolism are impacted during viral infection and since the presence of P2RX7 on mitochondrial surfaces of HEK293 hP2RX7 and N13 microglial cells has been shown recently by others (53, 54), we determined the mitochondrial activity in our feeder cells. As expected, maximal respiration and spare capacity were significantly increased in CM cells bearing the active P2X7 receptor as compared to CM WT or empty vector-transfected cells. Basal respiration was only increased in CM P2RX7 cells when compared to CM pcDNA3 cells. Also after infection, the maximal respiration and spare capacity were significantly upregulated in CM P2RX7 cells (**Figure 5B**). In addition, the extracellular acidification rate (ECAR), an indicator for glycolysis processes (55), was significantly higher in both, mock-infected and MVA-infected CM P2RX7 cells, as compared to CM WT or CM pcDNA3 cells (**Figure 5C**). Interestingly, intracellular ATP concentrations in the uninfected CM P2RX7 cells were comparable to CM WT cells but slightly lower than in CM pcDNA3 cells. After infection, however, intracellular ATP was significantly increased in CM P2RX7 cells only (**Figure 5D**), while extracellular ATP inversely correlated after infection showing a significant decrease for CM P2RX7 cells only (**Figure 5E**). In summary, we report that presence of P2RX7 significantly alters the energy metabolism of the cells by increasing the availability of mitochondrial ATP after MVA infection.

## Discussion

Studies have demonstrated the significant role of the P2X7 receptor in response to viral infections, exhibiting both protective and pathological functions (56). However, the function of P2RX7 during MVA infection and its relevance for antigen cross-presentation has not been investigated yet. Our findings suggest that the presence of an active P2X7 receptor in MVA-infected feeder cells can lead to a strongly increased antigen-presentation capacity by cross-presenting dendritic cells. This highlights a new function of purinergic receptor signaling in feeder cells serving as antigenic source for cross-presentation by dendritic cells.

Before delving into the specific role of the P2X7 receptor, we ensured its lack of function in the CM feeder cells (**Figure 1A**, **Supplementary Figure 1C**). As expected and as described in the literature, there was no discernible increase in intracellular Ca^2+^ in these cells, despite the stimulus with high concentrations of Bz-ATP, an agonist of P2X7 receptors (42, 43). Interestingly, when CM cells were treated with ATP, we could observe a desensitizing peak, which was ATP-dependent and strongly indicated the activation of other P2 receptors (43). However, when we reconstituted CM cells with the wild type, fully ATP-sensitive P2RX7 derived from BALB/c mice, we observed an increase in intracellular Ca^2+^, which was reversible upon treatment with A740003, a P2RX7-specific inhibitor (57). It also appeared that P2RX7 may play an important role during the initial phase of MVA infection, as infection for 4h resulted in a slight increase in intracellular Ca^2+^ values (**Supplementary Figure 1G**), while infection for 20h left intracellular Ca^2+^ levels unaffected (**Figure 1C**).

Based on our studies, MVA infection regulates P2RX7-specific functions, since the release of extracellular particless, which has been reported to be at least partly P2RX7-dependent (7, 8), was enhanced after MVA infection in the presence of an active P2RX7 (**Figures 1D, E**). We anticipate, however, that wildtype or empty vector-transfected CM cells with inactive P2RX7 would release apoptotic bodies instead of EPs, as MVA has been shown to trigger the initiation of apoptosis pathways in infected cells (11, 27). Further CM cell-specific studies are underway to understand the molecular pathways by which P2RX7 signaling modulates EP release mechanisms.

The presence of active P2RX7 in feeder cells during MVA infection led to a dramatic increase in CD8^+^ T cell activation and enhanced expression of SIINFEKL/H2-K^b^ complexes on murine antigen-presenting cells via cross-presentation (**Figures 2A, B**). This finding was corroborated in human cells by using a human feeder cell line, namely HEK293 cells, expressing a functional human P2RX7, while pre-treatment of CM P2RX7 with the P2RX7-specific inhibitor A740003 abrogated it (**Supplementary Figures 2A, B**). These results provide substantial evidence for the involvement of P2RX7 in feeder cells during MVA infection for antigen cross-presentation by dendritic cells. Since the presence of active P2RX7 in CM cells led to increased MHC II expression in dendritic cells (**Figure 2B** right) (58, 59), we hypothesize that P2RX7 alters the microenvironment e.g. by secretion of cytokines which stimulate non-infected bystander dendritic cells and has been previously suggested to be relevant for antigen presentation (60). Expression of P2RX7 has been associated with improved antigen presentation, especially due to the release of P2RX7-dependent extracellular particles containing inflammatory molecules and antigens (61, 62). Our data indicates a substantial P2RX7-dependent modulation of the production of MVA-derived antigen in feeder cells and its release or presentation to DCs enhancing MVA-mediated antigen cross-presentation.

Recent studies imply that feeder cells play a crucial role for antigen cross-presentation (33). In order to better understand this process, we decided to investigate the expression of viral antigens in feeder cells. Interestingly, viral particles were attached to the cell surface, but not internalized in CM P2RX7 cells as demonstrated by the increased viral particle load at 0hpi (**Figure 3B** left). In line with this finding, mRNA levels of all antigens tested (viral *B8R* and *A19L* as well as recombinant *Ova*) were initially lower in CM P2RX7 compared to CM WT cells, but were significantly increased at 20 hpi (**Figure 3A**) indicating that viral antigen expression kinetics is delayed in P2RX7 cells. This altered kinetics was corroborated by western blot analysis of OVA antigen synthesis in infected CM P2RX7 cells compared to the controls (**Figure 3C** left and middle). This implies that P2RX7 might play a role for viral entry, as previously described for other viruses such as HHV-6A and HBV/HDV (12, 63). Importantly, the MVA replication capacity in feeder cells was not altered in the absence or presence of P2RX7 resulting in comparable viral titers/multiplication rates (**Figure 3B** right) (19), thereby excluding that increased amounts of antigen due to increased viral replication in CM P2RX7 cells altered the cross-presentation capacities of APCs.

APCs require cell-associated antigens to be phagocytosed and processed for antigen cross-presentation (28, 47). It has been shown that expression of P2RX7 activates Caspase-8-mediated apoptosis and leads to the exposure of phosphatidylserine at the cell surface (64, 65). In fact, our studies demonstrate high expression of active Caspase-8 (**Figure 3C** left/right) and an increase of late apoptosis in CM P2RX7 cells at early time (8hpi) after mock or MVA infection (**Figure 3D** right) in the presence of P2RX7 thereby enhancing the decoration of the cell with the early apoptotic marker phosphatidylserine. Interestingly, at later time (20hpi), only cells without functional P2RX7 showed expression of phosphatidylserine on the cell surface (early apoptotic) (**Figure 3D**). Phosphatidylserine at the cell surface is required for vaccinia virus including MVA to enter cells (66). Vaccinia virus entry is facilitated by ‘apoptotic mimicry’, hence by flagging phosphatidylserine on mature virions and identifying it as apoptotic debris for uptake (67). Additionally, vaccinia virus is able to transfer phosphatidylserine molecules from the lipid bilayer of cell membranes to increase its infectivity (68), likely because the exposure of phosphatidylserine on the cell surface may act as an ‘eat-me’ signal for phagocytes (69) which potentially explains the increased expression of the BMDC maturation marker CD40 when co-cultured with uninfected CM P2RX7 cells. EPs, which emerge from the cell membrane, incorporate membrane-specific molecules, including phosphatidylserine (11, 70). Rausch and colleagues have proposed that vesicles bearing phosphatidylserine can trigger CD8^+^ T cell activation (70). Interestingly, in an experimental setting where either EP- or sup-fractions serve as the only source of antigen for cross-presentation (**Figures 4E, F**), we observed an increased CD8^+^ T cell activation which was accompanied by enhanced antigen-specific peptide/MHC class I surface expression in cross-presenting DC (**Figure 4G**). The above findings suggest that expression of a functional P2X7 receptor in feeder cells not only modulates antigen cross-presentation in APCs at the protein level, as previously suggested (71), but also influences viral gene expression and viral entry in feeder cells as well as phagocytosis by BMDCs.

P2RX7-expressing cells feature an altered secretome e.g. released extracellular particles might contain antigens, cytokines and regulatory RNAs that are important for antigen-presentation (8, 37, 39, 61). Although the amount of OVA protein in isolated extracellular particles or in the supernatant of MVA-infected cells was comparable in the absence or presence of P2RX7 (**Figure 4B**), the composition and expression level of (pro)inflammatory cytokines and chemokines in the supernatant of P2RX7 expressing cells was significantly altered (**Figure 4C**, **Supplementary Figure 3A**) in the presence of P2RX7. These results support the importance of P2RX7 during the initial phase of MVA infection as well as at the later stage, when infected feeder cells are co-cultured with antigen-presenting cells and continuously supply the microenvironment with stimulatory molecules and enhance cross-presentation (7, 72). The EP fraction and, significantly stronger the supernatant fraction of CM P2RX7 cells increased CD8^+^ T cell activation and SIINFEKL/H2-K^b^ expression by cross-presenting BMDCs when added to mock-infected feeder cells (**Figures 4F, G**). This effect was less pronounced when these fractions were added to MVA-infected feeder cells (**Supplementary Figure 3B–E**). The feeder cells produce apoptotic bodies upon MVA infection, which may contain antigens for phagocytosis. Since these were not eliminated by our isolation method (46), we filtered the supernatant (0.2µM) to exclude apoptotic bodies in this fraction. We confirmed that the secretome of CM P2RX7 cells significantly contributed to improved antigen cross-presentation by BMDCs (**Figure 4H** left/middle). This effect could be attributed to both, the secreted pro-inflammatory cytokines as well as small vesicles such as exosomes or micro vesicles released due to the presence of P2RX7 (8, 73). Additional soluble as well as cell-associated factors from infected feeder cells may be needed to fully license DCs for enhanced cross-presentation, as the total MHCI expression of BMDCs remained unchanged upon the addition of filtered supernatant fractions (**Figure 4D** right).

The P2RX7 protein is known to be expressed on the plasma membrane as well as on intracellular membrane structures, suggesting that it may have multiple functions depending on the compartment within the cell. Sarti and colleagues have previously described the enhancement of mitochondrial metabolism by P2RX7 (53). We confirmed the intracellular presence of P2RX7 in our feeder cells (**Figure 5A**) as well as an increase in mitochondrial activity in the presence of P2RX7 in our MVA-infection model (**Figures 5B, C**). P2RX7 expression correlated with maximal respiration rate and spare capacity in MVA- and mock-infected cells, demonstrating enhanced ability of the cells to respond to stress (74). Furthermore, the extracellular acidification rate (ECAR) was significantly enhanced in the presence of active P2RX7, delineating the altered glycolysis pathway in these cells (**Figure 5C**), in line with previously reported glycolytic activity attributed to P2RX7 (75). These results suggest that P2RX7 is able to change the bioenergetics state of cells (76), with or without MVA infection. ATP is required for efficient vaccinia virus production (77). Importantly, we observed higher ATP levels within P2RX7 competent feeder cells which were significantly increased after MVA infection (**Figure 5D**). In contrast, basal secretion of ATP by these cells (extracellular ATP level) was less or comparable when infected with MVA, while cells with inactive P2RX7 released significantly higher amounts of ATP into the supernatant after MVA infection (**Figure 5E**). As shown before, cells infected with MVA undergo apoptosis. Since ATP is released during cell death processes (27, 78), we suggest in line with others that ATP regulation is P2RX7-dependent in BALB/c P2RX7-bearing feeder cells, but it is apoptosis-dependent in feeder cells lacking the fully functional P2RX7 (79). Further studies are required to analyze if the available ATP can act in an autocrine manner and reactivate P2X7 receptors of the feeder cell, as previously reported (80). It has been described that infection of viral pathogens may drive metabolic reprogramming to allow for adaptation of the cell to biosynthetic and energetic needs required for viral replication (81, 82). We demonstrate that cells expressing a functional P2RX7 seem to handle MVA infections better due to prolonged active cell metabolism and increased energy levels resulting in increased overall cell fitness and delayed apoptotic cell death. The lack of ethidium bromide pore opening (**Supplementary Figure 1G**), another potential characteristic of P2RX7 plasma membrane expression (44), indicates a rather protective role of P2RX7 in feeder cells. In this respect, active Caspase-8 found in cells with functional P2RX7 seems to be activated in the absence of cell death, leading to the release of inflammatory cytokines and the restriction of pathogen growth (83). A signaling pathway associated with these processes and affected by P2RX7-dependent modulation may involve NF-κB (84).

In this study, we have uncovered significant factors influenced by the ATP-sensitive P2X7 receptor in MVA-infected feeder cells that promote antigen cross-presentation by dendritic cells. These factors include (i) the secretion of pro-inflammatory cytokines, (ii) delayed viral entry during MVA infection of CM P2RX7 cells associated with delayed viral gene expression and subsequent viral antigen synthesis. We further identified (iii) the release of small vesicles (<0.2µM) such as exosomes and microvesicles as well as viral mRNA containing EPs, as a potential source of viral antigens challenging the fact that only cell-associated antigens may be involved in the activation of CD8^+^ T cells by antigen cross-presentation. Additionally, we found that (iv) the presence of late apoptotic markers in feeder cells as well as (v) improved mitochondrial functions in feeder cells contribute to a favorable microenvironment for enhanced cross-presentation. Based on our results, we suggest that various signaling pathways triggered by active P2RX7 in infected CM feeder cells interplay and significantly contribute to the increased antigen cross-presentation capability of BMDCs leading to enhanced SIINFEKL//H2-K^b^ expression in BMDCs and subsequent CD8^+^ T cell activation.

### Limitations of study

Since the ECAR measured in this assay is only a quantitative measurement of the total amount of acid (H^+^) produced during both the tricarboxylic acid cycle and glycolysis, further assays may help to determine more specific alterations in the glycolytic metabolism (55). This study did not include an in-depth analysis of different extracellular particle fractions such as apoptotic bodies, microvesicles and exosomes. Further fractionation and subsequent characterization will allow to determine the exact content of these particles. Further work should address the role of DBA P2RX7 in CM cells. Even though the plasma membrane receptor is not functional in cells with a DBA background due to the P451L mutation (42), further studies may try to characterize other functions of this receptor during MVA infection *in vitro* and *in vivo*. Up to now, animal models such as appropriate P2RX7 knockout mice are lacking to investigate the role of the receptor *in vivo*. Since ATP may play a role during the co-culture of immune cells (85), future studies should assess its role in feeder cells, APCs and during co-culture. In addition, understanding how the altered mitochondrial metabolism affects EP release and composition as well as cross-presentation on the molecular level would be important for future studies. Due to the limitations of our *in vitro* murine model for the cross-presentation of MVA antigens, future research should include human cells.

## Materials and methods

The identifiers of all reagents and resources used are listed in **Supplementary Table 1** (**Supplementary Material**).

### Mice

For isolation of bone marrow female 12-to 16-week adult C57BL/6N mice were purchased from Janvier and were allowed to acclimate for a minimum of one week in the in-house animal facility. For weekly T cell stimulation, the spleen of adult C57BL/6N mice was used. Animals were maintained at the Zentrale Einrichtung für Tierversuchsanstalt (ZETT) at the University of Düsseldorf under specific pathogen-free conditions. Experimental procedures have been approved by the regional authorities (North Rhine-Westphalia State Environment Agency - LUA NRW, Germany) and the animal use committee at the University of Düsseldorf (Reg. No O119/11).

### Viruses

Recombinant MVA were generated by homologous recombination as previously described (33, 86). All stock preparations of MVA used in this study were diluted to a concentration of 1x10^9^ viral particles/mL and maintained at -80°C. Viral aliquots were thawed in a water bath, sonicated for one minute, briefly vortexed and spun down for usage. Freeze/thawed aliquots were not used more than three times.

### Infection of cells

Unless differently stated, cells were harvested, pelleted in a falcon and infected with MVA (MOI1, unless otherwise specified) for one to two hours at 37°C, 5% CO_2_ with intermittent shaking every 15min. Cells were washed twice before incubation with other cells for cross-presentation experiments. For the remaining experiments cells were seeded and incubated immediately. Since harvesting at different time points was required for expression kinetics analyses and titration experiments, cells were seeded and allowed to adhere before infection. Infection was then performed directly on the plate with intermittent shaking and washing after one hour of incubation at 37°C and 5% CO_2_. For infection in 96-well plates (Mitochondrial function assay and intracellular ATP determination assay) virus was added to each well of the plate, shaken every 15min for 1h and subsequently incubated for the remaining time frame at 37°C and 5% CO_2._

### Fluorometric analysis of intracellular Ca^2+^ levels or EtBr-guided pore opening

1x10^6^ cells were either mock- or MVA (MVA-PK1L-OVA) infected at an MOI of 1 for either 4h or 20h. For Ca^2+^ measurements infected cells were placed in a falcon for loading with 4µM FURA-2 AM (Sigma-Aldrich) in saline solution (12.5mM NaCl, 0.5mM KCl, 0.1mM MgSO_4_, 2mM HEPES, 0.55mM D-glucose, 0.5mM NaHCO_3_ (all Sigma-Aldrich)) supplemented with 0.5mM CaCl_2_ (pH 7.4, Merck) and 250µM sulfinpyrazone (Sigma-Aldrich) at 37°C for 20min. Cells were then washed, resuspended in saline solution and stimulated with the indicated concentrations of Bz-ATP (Sigma-Aldrich) and 1µM ionomycin (Invitrogen) for the recording of intracellular Ca^2+^ release. For detection of pore opening at the cell membrane, cells were loaded with 2µL ethidium bromide (EtBr (Sigma-Aldrich)) and stimulated with 200µM Bz-ATP and 100µM digitonin (Sigma-Aldrich). Measurements were done in a thermostat quartz cuvette using a Perkin-Elmer KS50 rotating and heating system at a wavelength of 340/380nm (excitation) and 505nm (emission) for intracellular Ca^2+^ release and at a wavelength of 360nm (excitation) and 580nm (emission) for EtBr- pore opening assay.

### Generation of stably transfected cell lines

Cloudman S91 cells (ATCC CCL-53.1) were seeded at a density of 1.5x10^5^ cells per well in a 6-well plate and transfected with either 3µG pcDNA3 control or P2RX7 encoding plasmid DNA using Lipofectamine reagent (Invitrogen) according to the manufacturer’s instructions. Briefly, plasmid DNA was dissolved in medium, incubated with 1µL PlusReagent for 5min and then 3µL Lipofectamine was added and further incubated for 30min at room temperature. The Lipofectamine-DNA mixture was then added dropwise to the cells and cells were selected for geneticin (0.2mg/mL) resistance two days post-transfection. Transfection efficacy was confirmed by Western Blot analysis of P2RX7 synthesis and by fluorometric analysis measuring the P2RX7-dependent intracellular Ca^2+^ increase.

### Live cell confocal imaging

Cells were grown on a round cover dish placed in a 6-well plate at a density of 5x10^5^ cells per well. Cells were stained with 2µM PKH-26 (Sigma-Aldrich) and 2µM Quinacrine (Sigma-Aldrich) for 10min at 37°C and 5% CO_2_ in saline saccharose solution (30mM saccharose, 0.1mM K_2_HPO_4_, 0.1mM MgSO_4_, 0.5mM D-glucose, 0.2mM HEPES (all Sigma-Aldrich)) supplemented with CaCl_2_ (pH7.4). Cells were placed in a holder device for round cover glasses and stimulated with 200µM Bz-ATP. Images were acquired in 6-second intervals for approximately 15min. Images were taken at 60x magnification of the Olympus Fluoview FV3000 (Olympus). Data visualization was achieved using OMERO software (Open microscopy imaging).

### Generation of bone marrow-derived dendritic cells

Bone marrow was obtained from 12-to 16-week-old C57BL/6N and 5x10^6^ bone marrow cells were seeded with 10% GM-CSF (obtained from supernatant of B16 cells expressing GM-CSF, originally kindly provided by Georg Häcker, Freiburg) in RPMI-medium (Gibco) containing 10% heat-inactivated FBS and 50µM 2-mercaptoethanol (M2 Medium) in 10cm Petri-dishes. On day three fresh M2 Medium and GM-CSF was added to the primary culture and on day six 10mL medium was replaced with fresh M2 Medium containing GM-CSF. BMDCs were used on day seven for all experiments.

### T cell restimulation

CD8^+^T cell lines were generated as described recently (33). For weekly T cell stimulation, both EL4 cells (ATCC TIB-39) and naïve splenocytes from C57BL/6N were irradiated with 100Gy or 30Gy, respectively. EL4 cells were loaded with 1µg/mL B8R-peptide (TSYKFESV; immunodominant peptide derived from the B8 protein from vaccinia virus) or Ova-peptide (SIINFEKL; derived from ovalbumin) and then co-incubated with splenocytes, CD8^+^ specific T cells and M2 Medium containing 5% TCGF (T-cell growth factor). Both peptides are H2-K^b^-restricted.

### Cross-presentation assay

Cloudman S91 murine melanoma (CM) cells (MHC I haplotype H2-d) were used as feeder cells for antigen cross-presentation assays. A total of 2x10^6^ cells were either mock- or MVA-PK1L-OVA (MOI1) infected for 20h, washed and subsequently incubated with psoralen (1µg/mL) (Sigma-Aldrich) for 15min at 37°C and 5% CO_2_ and treated with UV-A light (PUVA) for further 15min. Cells were harvested, transferred to a falcon and washed with medium. CM feeder cells were co-incubated with uninfected BMDCs, which were previously generated from bone marrow of C57BL/6N mice (MHC I haplotype H2-K^b^) at a ratio of 1:1 in a 6 cm dish for 18h. The next day, the co-culture of CM and BMDCs was harvested, washed in M2 Medium and resuspended in M2 medium in a final volume of 1mL. One part of the co-culture suspension (200µL) was immediately stained for the surface expression of peptide/MHCI complexes (SIINFEKL peptide within H2-K^b^) on BMDCs, while 100µL of the CM-BMDCs co-culture (containing 2x10^5^ BMDCs as antigen-presenting cells) was further incubated with 2x10^5^ B8R- or Ova- specific CD8^+^ T cells in the presence of 1µg/mL Brefeldin A (Sigma Aldrich) for 4h at 37°C and 5% CO_2_. Further analysis of CD8^+^ T-cell activation is described below.

### Intracellular cytokine staining (ICS)

To determine the antigen presentation capacity of dendritic cells in the cross-presentation setting, peptide-specific T cell lines were used as a read out system. After 4h incubation (see above cross-presentation assay), cells were washed with PBS and dead cells were excluded by staining with Fixable viability dye eFluor 506 (Invitrogen) (1:600) for 20min on ice. Cells were washed with FACS buffer (PBS supplemented with 1% BSA and 0.02% sodium azide) and then stained using anti-mouse CD8α eFluor 450 (eBioscience) (1:300) for 20min on ice. Subsequently, cells were permeabilized with BD Cytofix (BD Biosciences) for 15min on ice and then stained with Anti-mouse IFNy APC (Invitrogen) (1:400) and Anti-mouse TNFα PE-Cyanine7 (Invitrogen) (1:300) in 1:10 diluted BD Perm/Wash for 30min on ice. Cells were washed twice and resuspended in 1% PFA for subsequent analysis using the FACS Canto II device (BD Biosciences).

### SIINFEKL/H2-K^b^ surface staining/MHC II maturation staining

Antigen processing and presentation capacity was also assessed by measuring the MHCI/peptide complex formation as SIINFEKL/H2-K^b^ expression on the surface of the dendritic cells. For this co-cultured BMDCs (see above cross-presentation assay) were washed with PBS and dead cells were stained with Fixable viability dye eFluor 660 (Invitrogen) (1:2000) for 20min on ice. Fc-receptors were blocked using anti-mouse CD16/CD32 (eBioscience) (1:200). After Fc-blocking, surface staining was performed for 30min on ice using anti-mouse CD11c PE (Invitrogen), anti-mouse H2-Kb FITC (Biolegend) and anti-mouse SIINFEKL/H2-K^b^ PE-Cyanine 7 (eBioscience) (all 1:300 in FACS buffer). Cells were washed twice and resuspended in 1% PFA for subsequent analysis using FACS Canto II. Alternatively, cells were stained with Fixable viability dye eFluor 506 (Invitrogen) (1:600) for 20min on ice, followed by the Fc-blocking step and surface staining with CD11c APC-Cyanine 7 (BD Pharmingen) and MHCII PE (all 1:300 in FACS buffer).

### Viral or cellular gene expression

For kinetic analysis of viral gene expression (0h to 24hpi), 2x10^6^ cells were infected with MVA-PK1L-Ova (MOI1) for 1h at 4°C, resulting in the virus attachment to the cell surface. After washing cells were harvested at the indicated time points, spun down and the pellet was resuspended for total RNA isolation as described in the manufacturer´s protocol (RNeasy Mini Kit (Qiagen)). Briefly, cells were lysed using RLT buffer containing 10µL 2-ß-mercaptoethanol and mixed with one volume of 70% ethanol for subsequent isolation using the RNeasy Mini spin column. cDNA was then transcribed using the Revert Aid H minus first strand cDNA synthesis (Thermo Fisher Scientific) according to the manufacturer’s instructions and used as a template for subsequent quantitative PCR reaction with PowerUp SYBR Green Master Mix (Applied Biosciences). Expression of viral *B8R*, *Ova*, *A19L* and cellular *P2rx7* genes was normalized to expression of *18S-rRNA* housekeeping gene and ΔΔCT was calculated by further comparison of ΔCT values with the 0h time point of CM wildtype cells. Primer sequences are listed in **Supplementary Table 1**.

### Viral replication

In order to determine the replication capacity of MVA, 1x10^6^ CM cells (WT, pcDNA3- or P2RX7- transfected) were infected with MVA-p7.5-GFP at MOI5 for 0h (1h at RT), washed and further incubated until harvested at 4h, 8h or 24hpi. Collected samples were vortexed and subjected to three rounds of freeze-thaw-sonication cycles to release viral particles. Viral suspensions were then used to prepare serial dilutions that were plated on 96-well plates containing MVA-permissive DF-1 cells (ATCC CRL-12203) (80% confluent). Fluorescent signal and cytopathic effect was monitored for seven days post-infection to determine the 50% endpoint titer of viral particles per milliliter by using the Spearman-Karber method to calculate the tissue culture infectious dose 50 (TCID_50_).

### Western Blot analysis

For extraction of proteins, 2x10^6^ cells were infected (see above “infection of cells”) and harvested at the indicated time points. Collected cells were spun down by centrifugation, washed with PBS and resuspended in RIPA buffer (Thermo Fisher Scientific) containing HALT Protease & Phosphatase Inhibitor cocktail (Thermo Fisher Scientific) (1:100). After three rounds of freeze-thaw-sonication cycles, supernatants were harvested after a single centrifugation step at full speed for five minutes at 4°C. Protein content was quantified using the Pierce BCA Protein Assay Kit (Thermo Fisher Scientific). SDS-PAGE and blotting on nitrocellulose membranes was performed as described elsewhere (87). Membranes were incubated with Anti-Ovalbumin (Rockland) (1:20 000); Anti-Cleaved caspase-8 (Cell signaling) (1:1000); Anti-P2RX7 (Sigma-Aldrich) (1:200) and Anti-ß- Actin (Sigma-Aldrich) (1:50 000). Relative quantification of specific proteins was done by calculating ratio of the protein of interest with the ß-Actin loading control using the ImageJ analysis tool (US National Institutes of Health, Bethesda, USA).

### Phosphatidylserine exposure analysis

Surface staining of phosphatidylserine residues on MVA-infected cells was done according to Apotracker-Green protocol (Biolegend) at either 6hpi or 20hpi. Briefly, 2x10^5^ cells were washed with FACS buffer and incubated in 400nM Apotracker-Green staining solution for 20min at room temperature. Cells were subsequently stained with fixable viability dye eFluor 660 (1:2000) for 20min on ice, washed and immediately analyzed by FACS. Cells were either gated for APC-negative (non-permissive for viability dye) and FITC-positive (Apotracker Green-positive) populations, designating early apoptotic cells or gated for APC- and FITC-double-positive populations, indicating late apoptotic cells.

### Isolation of extracellular particles and supernatant fractions

For extracellular particle isolation the protocol was adapted according to Pegoraro and colleagues (8). Four T75 flasks (approximately 8x10^6^ cells/flask) were seeded with CM cells one day before infection to obtain 90% confluency. Before infection, one flask per cell line was counted in order to calculate the respective MOI. Cells were allowed to rest for 30min at room temperature and after washing 3mL medium was added in each flask. MVA-PK1L-Ova (MOI 1.5) was added and flasks were shaken every 15min for one hour (at 4°C for RNA isolation). After one hour cells were washed and harvested (0h value) or further incubated for a total of 20h at 37°C and 5% CO_2_. For harvesting, medium was discarded, cells were washed with PBS and 3mL saline solution supplemented with 0.05mM CaCl_2_ was added. Cells were stimulated with 200µM Bz-ATP for 30min at 37°C. Thereafter, the supernatant was aspirated and centrifuged at 300g for five minutes at 4°C to remove cell debris. The cleared supernatant was harvested, aliquoted in Eppendorf tubes and centrifuged at 20.000 g for one hour at 4°C. The supernatant was discarded and the remaining extracellular particle fraction (EP-fraction) was either used for quantitative RNA analyses (resuspended in RLT buffer with 2-ß-mercaptoethanol), western blot analyses (resuspended in RIPA buffer with HALT Protease & Phosphatase Inhibitor cocktail) or for cross-presentation assays (resuspended in PBS). For cross-presentation assays, EP fractions were additionally PUVA treated prior to the last centrifugation step, as described above.

For isolation of supernatants, 2x10^6^ cells per condition tested were used. Cells were either MVA-PK1L-Ova (MOI1) or mock-infected for the indicated time (8h or 20h for western blot analysis; 20h for Legendplex and cross-presentation assays), harvested and supernatants (sup-fraction) were collected after centrifugation at 300g for five minutes. For indicated experiments, supernatant fractions were further passed through a 0.2µM size pore filter (fil sup-fraction) to be used for cross-presentation experiments. All supernatant fractions (sup- or fil sup-fractions) used for cross-presentation assays were additionally PUVA treated as described above.

### Cytokine and chemokine analysis in supernatants

The release of cytokines/chemokines was analyzed using the Legendplex MU anti-virus response panel (Biolegend). Briefly, 2x10^6^ cells were either MVA-PK1L-Ova (MOI1) or mock-infected for 20h. After harvesting the cell suspensions, supernatants were collected after centrifugation at 300g for 5min and processed according to the manufacturer’s instructions. Data was analyzed using the Biolegend LEGENDplex Data Analysis Software (Biolegend).

### Cross-presentation assays using EP- or supernatant-fractions

Infection of CM feeder cells was performed as described above for cross-presentation assays. On day two, CM WT feeder cells (either mock- or MVA-PK1L-OVA infected, MOI1) were co-incubated with BMDCs and, additionally, pulsed with either EP-, sup- or fil sup-fractions. These fractions were isolated from either infected CM pcDNA3 (transfected cells with inactive P2RX7) or infected CM P2RX7 cells (transfected cells with active P2RX7) after 20hpi as described above. On day 3, cross-presentation assays were continued as described above.

### Intracellular quantification of P2RX7

To assess the expression of P2RX7, 2x10^5^ CM cells (WT, pcDNA3- or P2RX7-transfected) were mock- or MVA-PK1L-OVA (MOI1) infected. After 20hpi, cells were stained with fixable viability dye eFluor 660 (1:2000) for 20min on ice, permeabilized with BD Cytofix for 15min on ice and then stained with anti-P2RX7 (1:200) for one hour on ice to quantify the intracellular presence of P2RX7. Cells were further incubated with anti-mouse-IgG-PE (Jackson laboratories) (1:200) secondary antibody for 30min on ice, washed and immediately used for FACS analysis by FACS Canto II.

### Mitochondrial metabolism analysis

The day prior to infection, 2x10^4^ cells were seeded in a Seahorse XF96 Cell culture Microplate (Agilent Technologies). Cells were allowed to adhere for 1h at room temperature and were further incubated at 37°C at 5% CO_2_ overnight. The next day cells were infected with MVA-PK1L-Ova (MOI5, 6h). Mitochondrial function was assessed using the Seahorse XF Cell Mito Stress test (Agilent technologies) according to the manufacturer’s instructions. Compounds have been used at the concentration of 15µM for Oligomycin, 5µM for FCCP and 5µM for Rot/AA.

### Intracellular ATP measurements

The day prior to infection, 5x10^4^ cells were seeded in a 96 flat well chimney base plate and incubated overnight at 37°C and 5% CO_2_. Cells were infected with MVA-PK1L-Ova for 6h (MOI5) before intracellular ATP concentrations were determined using the Luminescent ATP detection assay kit (Abcam) as described in the supplier´s protocol. Briefly, cells were lysed and ATP was stabilized by a detergent during a shaking step. After the addition of the substrate solution, prompted luminescence was measured and compared to ATP standard samples using a Spark plate reader (Tecan).

### Extracellular ATP measurement

1x10^6^ CM cells (WT, pcDNA3- or P2RX7-transfected either MVA-PK1L-Ova or mock-infected, MOI5) were seeded in 1mL in a 6-well plate. The supernatant was harvested after 6hpi. For each condition, 50µL supernatant was incubated with 50µL of FirezymeB Diluent buffer (Firezyme). Samples as well as an ATP standard (Sigma- Aldrich) were compared in a standard curve at serial dilutions run by the Luminometer Victor 3 1420 Multiwell counter (Perkin Elmer) with automated addition of 100µL Enliten Luciferase/Luciferin reagent (Promega) to detect emitted luminescence.

### Quantification and statistical analysis

Details on statistical analyses are integrated in figure legends. When indicated, data was normalized to untreated or WT control cells. Unpaired two-tailed student’s t-test was used to calculate statistical significances using Prism 8 (GraphPad Software). Extracellular particles (**Figure 1C**) were quantified by counting three adjacent frames of each replicate upon Bz-ATP stimulus and normalized to cell numbers (determined by quinacrine staining) per frame. Graphical data represent mean values with error bars indicating SD or SEM with P-values of ≤ 0.05 (*), ≤ 0.01 (**), ≤ 0.001 (***) and ≤ 0.0001 (****) indicating significant differences between groups.

## Data availability statement

The original contributions presented in the study are included in the article/**Supplementary Material**. Further inquiries can be directed to the corresponding author.

## Ethics statement

Ethical approval was not required for the studies on humans in accordance with the local legislation and institutional requirements because only commercially available established cell lines were used. The animal study was approved by North Rhine-Westphalia State Environment Agency - LUA NRW, Germany) and the animal use committee at the University of Düsseldorf (Reg. No O119/11). The study was conducted in accordance with the local legislation and institutional requirements.

## Author contributions

YL: Writing – review & editing, Data curation, Formal analysis, Investigation, Validation, Writing – original draft. SM: Investigation, Writing – review & editing. GA: Investigation, Writing – review & editing. JW: Investigation, Methodology, Writing – review & editing. IK: Investigation, Methodology, Writing – review & editing. EDM: Investigation, Methodology, Writing – review & editing. AP: Investigation, Methodology, Writing – review & editing. RL: Methodology, Writing – review & editing. KK: Data curation, Formal Analysis, Investigation, Methodology, Validation, Writing – review & editing. PP: Data curation, Formal Analysis, Investigation, Methodology, Validation, Writing – review & editing. RT: Investigation, Methodology, Writing – review & editing. FDV: Methodology, Writing – review & editing. EA: Methodology, Supervision, Validation, Writing – review & editing. ID: Conceptualization, Data curation, Funding acquisition, Methodology, Project administration, Resources, Supervision, Validation, Writing – original draft, Writing – review & editing.
