# Supplementary material for: The purinergic receptor P2X7 as a modulator of viral vector-mediated antigen cross-presentation
Source: Front Immunol. 2024 Apr 22;15:1360140. doi: 10.3389/fimmu.2024.1360140 (PMC11070468; doi:10.3389/fimmu.2024.1360140)
Supplement: Supplementary file 1 [file DataSheet_1.docx]

**Supplementary Information**

**The Purinergic receptor P2X7 as a modulator of viral vector-mediated antigen cross-presentation**

Ylenia Longo, Sara Moreno Mascaraque, Giuseppe Andreacchio, Julia Werner, Ichiro Katahira, Elena De Marchi, Anna Pegoraro, Robert Jan Lebbink, Karl Köhrer, Patrick Petzsch, Ronny Tao, Francesco Di Virgilio, Elena Adinolfi, Ingo Drexler


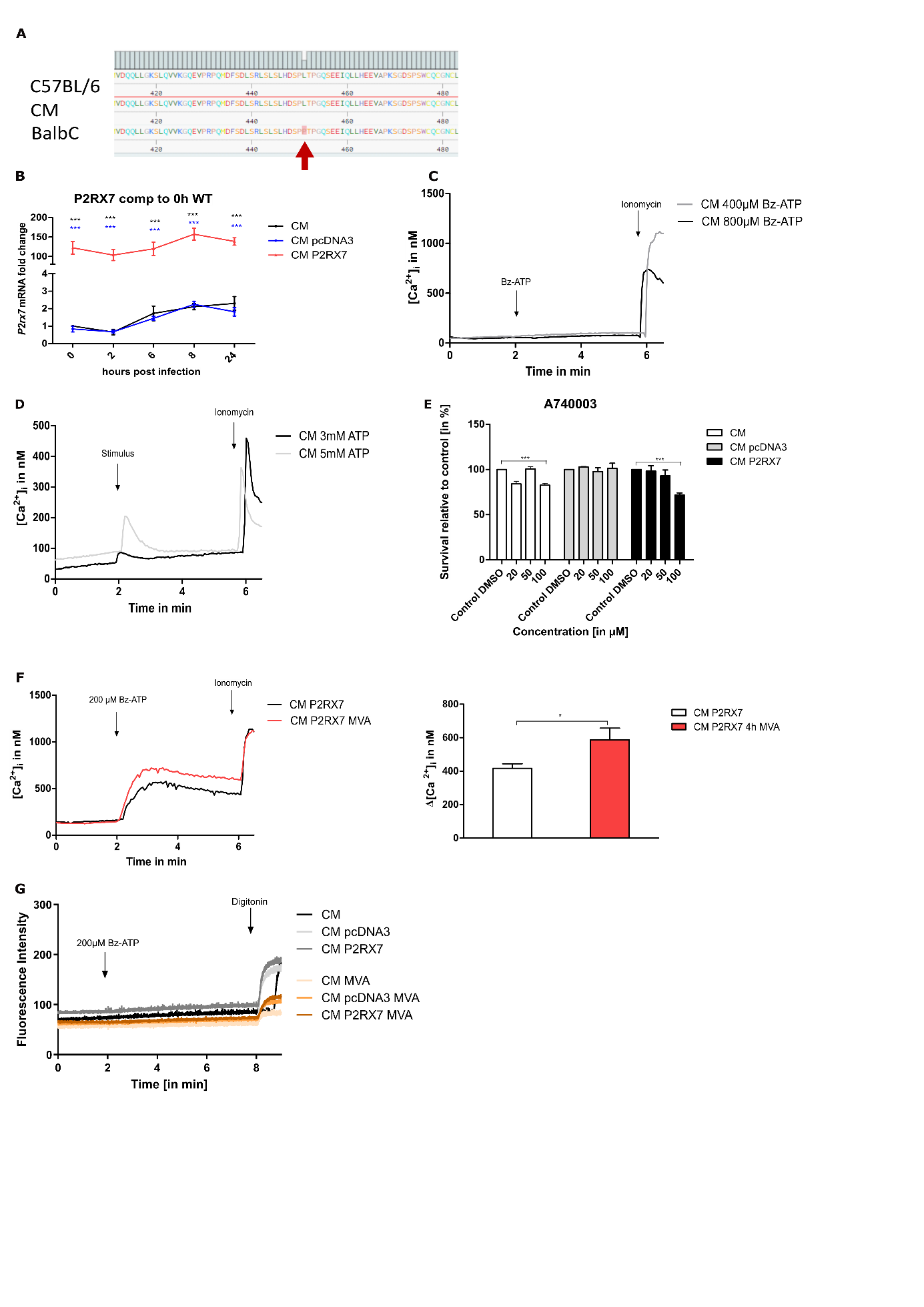


**FIGURE S1 I** **Characterization of purinergic receptors in CM cells after MVA infection***.* (A) Sequencing analysis of the region with P451L mutation (42) in CM WT cells (DBA/2 mouse strain) compared to homologous sequences in C57BL7/6 mice (GenBank: AJ489296.1) or BALB/c mice (GenBank: AJ489297.1). (B) *P2rx7* gene expression kinetics was assessed from 0h to 24hpi with MVA-PK1L-Ova expressing ovalbumin under control of the vaccinia virus early promoter PK1L at MOI1 in CM WT (CM), empty vector (CM pcDNA3), or P2RX7 (CM P2RX7) transfected cells and mRNA fold change was calculated by comparison to the 0h time point of CM WT cells. (C) CM WT cells were loaded with FURA-2-AM fluorescent indicator and then stimulated with the indicated concentrations of Bz-ATP to assess P2RX7-specific activity by intracellular calcium influx. (D) CM WT cells were loaded with FURA-2-AM and then received a stimulus with the indicated concentrations of ATP to asses the activity of other purinergic receptors by measurement of intracellular calcium concentrations. (E) *In vitro* toxicity assay using MTT indicator in CM, CM pcDNA3, or CM P2RX7 transfected cells using different concentrations of the P2RX7-specific inhibitor A740003 for 20 hours treatment. (F) (Left) Fluorimetric analysis of intracellular calcium concentrations in CM P2RX7 transfected cells upon 4h infection with MVA at MOI1 or mock infected and subsequent treatment with Bz-ATP and Ionomycin. (Right) Calculated Δ between the peak and the basal values of each condition shown in (F). (G) Ethidum bromide pore opening was measured upon loading of mock-or MVA-PK1L-Ova infected (MOI1, 20hpi) CM, CM pcDNA3 or CM P2RX7 cells with ethidium bromide and stimulation with 200µM Bz-ATP and digitonin. Values shown are mean with SD or SEM (B, E and G) of n=3 independent or (A,C,D) n=1-2 experiments with statistical significance (P) *P ≤ 0.05 **P ≤ 0.01; ***P ≤ 0.001.


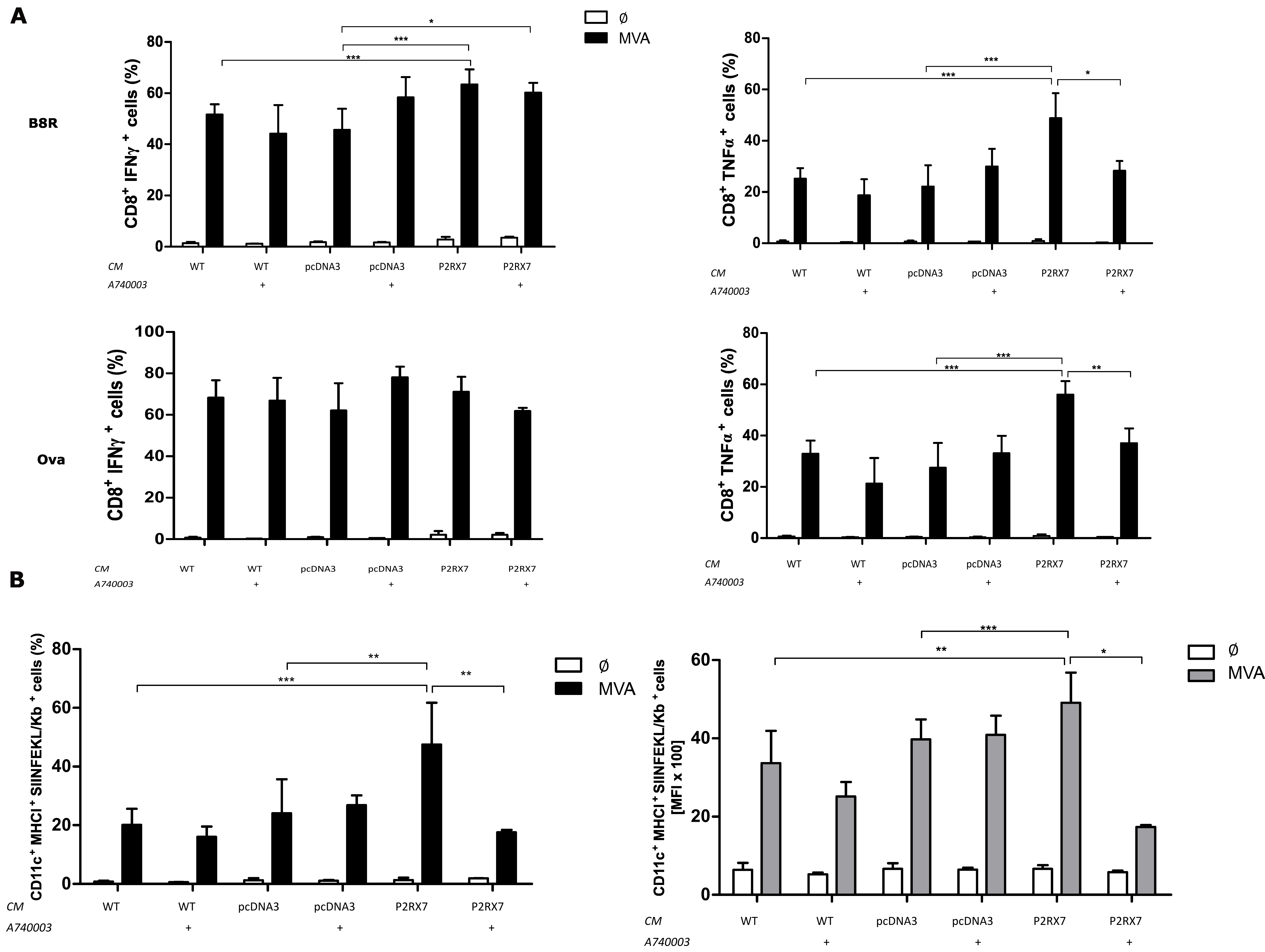


**Figure S2 I *CD8+ T cell activation and SIINFEKL/H2Kb surface expression is reduced when active P2RX7 is inhibited in infected feeder cells.*** (A) CM WT (CM), empty vector (CM pcDNA3), or P2RX7 (CM P2RX7) transfected cells were pre-treated for one hour with 20µM A740003, a specific inhibitor of P2RX7, under shaking conditions. After washing, cells were mock (Ø) or MVA-PK1L-OVA infected at MOI1 in the presence of A740003 for 20h. Infected cells were subsequently PUVA-treated, washed and co-cultured with uninfected BMDCs for 18h. (A) CD8^+^ T cells were added for 4h and (upper) B8R or (lower) OVA-specific T cell activation was measured by intracellular cytokine staining of IFNy or TNFα. (B) SIINFEKL/H2Kb surface expression in co-cultured BMDCs with mock or MVA-PK1L-OVA infected CM cells. Frequency (left) and MFI (right) are shown. All experiments are presented as mean with SD of at least n=3 independent with statistical significance (P) with *P ≤ 0.05 **P ≤ 0.01; ***P ≤ 0.001.

**
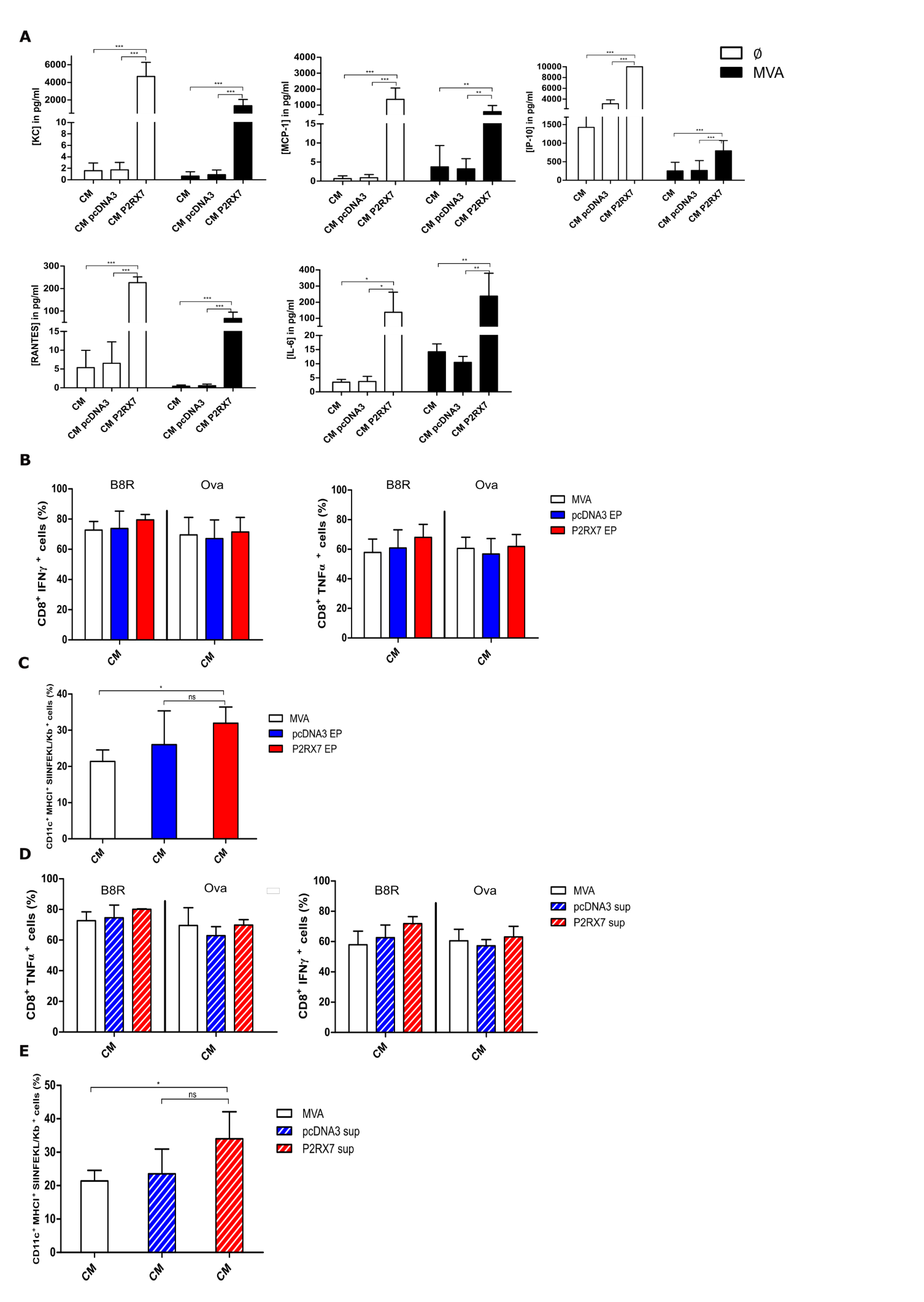
**

**Figure S3 I (A) *Presence of functional P2RX7 in CM cells allows for a significantly altered cytokine and chemokine repertoire*.** CM WT (CM), empty vector (CM pcDNA3) or P2RX7 (CM P2RX7) transfected cells were either mock (Ø) or MVA-infected (MVA) at MOI1. After 20hpi supernatants were analyzed for the presence of cytokines/chemokines using Legendplex assay. **(B, C) Extracellular particle fractions or (D, E) supernatant fractions from P2RX7 transfected cells do not alter CD8+ T cell activation but increase SIINFEKL/H2-K^b^ surface expression when added to infected CM cells.** Extracellular particles or the supernatant fraction were isolated from CM P2RX7 or CM pcDNA3 cells after MVA-PK1L-OVA (MOI1.5 or MOI1, respectively, 20hpi) and added to either mock (Ø) or MVA-PK1L-Ova (MVA) (MOI1, 20hpi) infected CM cells. Cells were then washed, virus PUVA inactivated and co-cultured with BMDCs for 20h. (B,D) The next day B8 or Ova specific CD8^+^ T cells were added to the CM-BMDC mixture and incubated for 4h in the presence of BFA. IFNү or TNFα expression by B8R- or OVA-specific CD8^+^ T cells was determined by ICS followed by FACS. (C,E) SIINFEKL/H2-K^b^ surface expression upon co-culture with uninfected BMDCs derived from (C) the EP-fraction or (E) sup-fraction stimulated setting. Data represent mean with SD of at least n=3 independent experiments with statistical significance (P) *P ≤ 0.05 **P ≤ 0.01; ***P ≤ 0.001.

**Supplementary Table 1**

| **REAGENT** | **SOURCE** | **IDENTIFIER** |
| --- | --- | --- |
| **Antibodies** | | |
| Quinacrine dihydrochloride | Sigma-Aldrich | Cat.No: Q3251 |
| PKH26 Red fluorescent cell membrane labeling | Sigma-Aldrich | Cat.No: P9691 |
| Anti-mouse IFNy APC | Invitrogen | Clone: XMG1.2 |
| Anti-mouse TNFa PE-Cyanine7 | Invitrogen | Clone: Mp6-XT22 |
| Anti-mouse CD11c PE | Invitrogen | Clone: N418 |
| Anti-mouse SIINFEKL/H2-K^b^ PE-Cyanine 7 | eBioscience | Clone: eBio25-D1.16 |
| Anti-mouse CD8a eFluor 450 | eBioscience | Clone: 53-6.7 |
| Anti-Ovalumin, rabbit | Rockland | Cat.No: 100-4133 |
| Anti-Cleaved caspase-8, rabbit | Cell signaling | Clone: D5B2 |
| Anti-ß- Actin, mouse | Sigma-Aldrich | Clone: AC-74 |
| Anti- P2RX7, rabbit | Sigma-Aldrich | Cat.No: P8232-2mL |
| Apotracker- Green | Biolegend | Cat.No: 427402 |
| Fixable viability dye eFluor 506 | Invitrogen | N/A |
| Fixable viability dye eFluor 660 | Invitrogen | N/A |
| a-mouse, IgG | Jackson laboratories | N/A |
| a-rabbit, IgG | Jackson laboratories | N/A |
| Anti-mouse H2-K^b^ FITC | Biolegend | Clone: AF6-88.5 |
| Anti-mouse CD16/CD32 | eBioscience | Clone: 93 |
| R-Phycoerythrin AffiniPure F(ab')₂ Fragment Goat Anti-Mouse IgG, Fcγ fragment specific (Anti-mouse-IgG-PE) | Jackson laboratories | Cat.No: 115-116-071 |
| **Virus strains** | | |
| MVA-PK1L-OVA  (MVA expressing Ovalbumin under the early promoter PK1L) | Ronny Tao, Institute of Virology UKD, HHU Düsseldorf | N/A |
| MVA-p7.5-GFP  (MVA expressing GFP under the early/late p7.5 promoter) | Ronny Tao, Institute of Virology UKD, HHU Düsseldorf | N/A |
| MVA-PK1L-mCherry-OVA  (MVA expressing mCherry-Ovalbumin fusion protein under the early promoter PK1L) | Ronny Tao, Institute of Virology UKD, HHU Düsseldorf | N/A |
| **Chemicals and peptides** | | |
| FURA-2 AM | Thermo Fisher Scientific | Cat.No: F1201 |
| Bz-ATP | Sigma-Aldrich | Cat.No: 112898-15-4 |
| Ionomycin | Invitrogen | Cat.No: J62448.MCR |
| B8R_20-27__peptide, H2-K^b^ restricted  TSYKFESV | peptides &elephants | 95% purity |
| Ova_257-264__peptide, H2-K^b^ restricted  SIINFEKL | peptides &elephants | 95% purity |
| NaCl, KCl, MgSO_4_, D-glucose, saccharose, NaH_2_PO_4_, HEPES, NaHCO_3_, K_2_HPO_4_ | Sigma-Aldrich | Standard products |
| Calcium chloride | Merck | Cat.No: 10043-52-4 10043-52-4 |
| Sulfynpyrazole | Sigma-Aldrich | Cat.No: S2159000 |
| ATP | Sigma-Aldrich | Cat.No: A6419-1G |
| A740003 | Tocris | Cat.No: R3701 |
| RPMI Medium 1640 (1x) + GlutaMax | Gibco | Cat.No: R6504 |
| FBS Supreme | Pan Biotech | Cat.No: P30-3031 |
| PBS | Gibco | Cat.No: 10010001 |
| Granulocyte-macrophage colony stimulating factor (GM-CSF) | Institute of Virology UKD, HHU Düsseldorf | Harvested from supernatant of B16-GM-CSF producing cells |
| Geneticin selective antibiotic | Gibco | Cat.No: 10131-035 |
| Ethidium bromide | Sigma-Aldrich | Cat.No: E1510-10ML |
| Digitonin | Sigma-Aldrich | Cat.No: D141 |
| Thiazolyl blue tetrazolium bromide (MTT) | Thermo Fisher Scientific | Cat.No: 158990050 |
| BD Pharm Lysing buffer | BD Biosciences | Cat.No: 15838518 |
| 4′-Aminomethyltrioxsalen-hydrochlorid (Psoralen) | Sigma-Aldrich | Cat.No: A4330-5MG |
| Brefeldin A | Sigma-Aldrich | Cat.No: B7651 |
| PowerUp SYBR Green Master Mix | Applied Biosciences | Cat.No: A25742 |
| Lipofectamine LTX reagent | Invitrogen | Cat.No: 15338-030 |
| FirezymeB Diluent buffer | Firezyme | N/A |
| Enliten Luciferase/Luciferin reagent | Promega | Cat.No: FF2021 |
| ProLong diamond antifade mountant | Invitrogen | Cat.No: P36961 |
| RIPA buffer | Thermo Fisher Scientific | Cat.No: 89900 |
| **Commercial assays** | | |
| Legendplex MU anti-virus response panel 13-plex | Biolegend | Cat.No: 740622 |
| Seahorse XF Cell Mito Stress test | Agilent technologies | Cat.No: 103015-100 |
| RNAse free DNAse set | Qiagen | Cat.No: 79254 |
| BD Cytofix/Cytoperm Fixation/Permeabilization kit | BD Biosciences | Cat.No: 554714 |
| BD Perm/Wash | BD Biosciences | Cat.No: 554723 |
| Revert Aid H minus first strand cDNA synthesis | Thermo Fisher Scientific | Cat.No: K1621 |
| HALT Protease & Phospatase Inhibitor cocktail, EDTA-free (100x) | Thermo Fisher Scientific | Cat.No: 78441 |
| Pierce BCA Protein Assay Kit | Thermo Fisher Scientific | Cat.No: 23227 |
| Luminescent ATP detection Assay Kit | abcam | Cat.No: ab113849 |
| RNeasy Mini Kit | Qiagen | Cat.No: 74106 |
| **Cell lines** | | |
| Cloudman S291 melanoma (Clone M-3) - CM | ATCC | CCL-53.1 |
| CM pcDNA3  (CM cells transfected with empty vector plasmid pcDNA3) | This paper | N/A |
| CM P2RX7  (CM cells transfected with P2RX7 plasmid) | This paper | P2RX7 plasmid from (1) |
| HEK293 | Elena Adinolfi, University of Ferrara | HEK293 cells from  (2) |
| HEK293 hP2RX7 | Elena Adinolfi, University of Ferrara | HEK293 cells from  (2) |
| CD8 -T cell line (specific for B8R or Ova) | Ronny Tao, Institute of Virology UKD, HHU Düsseldorf | N/A |
| DF-1 | ATCC | CRL-12203 |
| EL4 | ATCC | TIB-39 |
| **Organisms/strains** | | |
| Mouse: C57BL/6N | Janvier | N/A |
| **Oligonucleotides** | | |
| *B8R_primer*  Fw: ATC CGC ATT TCC AAA GAA TG  Rev: ACA TGT CAC CGC GTT TGT AA | Sha Tao, Institute of Virology UKD, HHU Düsseldorf | (3) |
| *Ova_ primer*  Fw: CAC AAG CAA TGC CTT TCA GA  Rev: GAC TTC ATC AGG CAA CAG CA | Sha Tao, Institute of Virology UKD, HHU Düsseldorf | (3) |
| *A19L_primer*  Fw: GCA TGA CGT GTT CTG CCT  Rev: GGC CAG TGT ATT ACC CCT CA | Sha Tao, Institute of Virology UKD, HHU Düsseldorf | (3) |
| *P2rx7_primer*  Fw: CAC ACC AAG GTC AAA GGC AT  Rev: CAC TTG GCC TTC TGA CTT GAC | This paper | N/A |
| *18srRNA_primer*  Fw: AAA CGC CTA CCA CAT CCA AG  Rev: CCT CCA ATG GAT CCT CGT TA | Sha Tao, Institute of Virology UKD, HHU Düsseldorf | (3) |
| **Recombinant DNA** | | |
| pcDNA3- Plasmid  (empty vector plasmid carrying the geneticin antibiotic resistance gene) | Elena Adinolfi, University of Ferrara |  |
| P2RX7- Plasmid  (plasmid encoding for P2RX7 from BalbC mice, carries the geneticin antibiotic resistance gene) | Elena Adinolfi, University of Ferrara | (1) |
| **Software** | | |
| OMERO | Open microscopy environment | N/A |
| ZEISS Zen miscroscopy software | Zeiss | N/A |
| FlowJo | Tree star Inc. | N/A |
| GraphPad Prism 8 | GraphPad, La Jolla California, USA | N/A |
| ImageJ | US National Institutes of Health, Bethesda, USA | N/A |
| Wave 2.4.3 | Agilent technologies | N/A |
| Benchling (Biology software) | Benchling (RRID:SCR_013955) | Retrieved from https://benchling.com (2023) |
| BD FACS Diva I | BD | N/A |
| 7500 Software v2.3 | Applied Biosystems | N/A |
| Cary Eclipse software | Agilent technologies | N/A |
| Biolegend LEGENDplex Data Analysis Software | Biolegend | N/A |
| BioRender | BioRender | Illustrations were created with BioRender.com |
| Morpheus | Broad Institute | Heat Maps were created with software.broadinstitute.org/morpheus |

**References (Supplementary Table 1)**

1. Sarti AC, Vultaggio-Poma V, Falzoni S, Missiroli S, Giuliani AL, Boldrini P, et al. Mitochondrial P2X7 Receptor Localization Modulates Energy Metabolism Enhancing Physical Performance. Function. 2021;2(2)
2. Adinolfi E, Cirillo M, Woltersdorf R, Falzoni S, Chiozzi P, Pellegatti P, et al. Trophic activity of a naturally occurring truncated isoform of the P2X7 receptor. FASEB J. (2010) 24:3393–404. doi: 10.1096/fj.09-153601
3. Tao S, Tao R, Busch DH, Widera M, Schaal H, Drexler I. Sequestration of late antigens within viral factories impairs MVA vector-induced protective memory CTL responses. Front Immunol. (2019) 10:2850. doi: 10.3389/fimmu.2019.02850
